# Effect of Reduced Fluoroquinolone Use on Cephalosporin Use, Susceptibilities and *Clostridioides difficile* Infections

**DOI:** 10.3390/antibiotics11101312

**Published:** 2022-09-27

**Authors:** Callyn Mariah Wren, Jill Cowper, Nickie Greer, Laurel Goldin, Alicia Perry

**Affiliations:** 1 HCA Healthcare and The University of Tennessee, Nashville, TN 37203, USA; 2 HealthTrust Supply Chain, Nashville, TN 37203, USA

**Keywords:** fluoroquinolone, cephalosporin, *Clostridioides difficile*, antimicrobial stewardship, antimicrobial resistance

## Abstract

Background: Overuse of fluoroquinolones has led to concerning rates of resistance, particularly among Gram-negative organisms. They are also highly implicated as a risk factor for *Clostridioides difficile* infection, and reports of other serious adverse events led to recommendations to restrict their use. Our health system began targeting the reduction in unnecessary fluoroquinolone prescribing in 2018, aiming to promote their safe and effective use. Broad-spectrum cephalosporins are often used as an alternative to fluoroquinolones. We sought to evaluate whether decreased fluoroquinolone use was associated with increased third- and fourth-generation cephalosporin use and whether these changes in utilization impacted other outcomes, including *C. difficile* infection (CDI) rates and susceptibilities among Gram-negative organisms. Methods: This retrospective descriptive analysis included adult patients who received a fluoroquinolone or broad-spectrum cephalosporin in a three-year time period across a large healthcare system. The primary objective was to evaluate the change in days of therapy (DOT) of fluoroquinolones and third- and fourth-generation cephalosporins. Secondary objectives included rates of resistance among common Gram-negative organisms, CDI, and analyses stratified by antibiotic indication. Results: Cephalosporin use increased by an average of 1.70 DOT/1000 PD per month (*p* < 0.001). Additionally, fluoroquinolone use decreased by an average of 1.18 DOT/1000 PD per month (*p* < 0.001). *C. difficile* infections decreased by 0.37 infections/10,000 patient-days per month (*p* < 0.001). Resistance to fluoroquinolones remained stable from 2018 to 2020, and a declining trend was observed in 2021. Conclusion: This study demonstrated that reduced fluoroquinolone use in a large healthcare system was associated with increased usage of broad-spectrum cephalosporins, decreased CDI and improvements in resistance patterns.

## 1. Introduction

The approval of fluoroquinolones led to widespread popularity due to their broad spectrum of activity and ease of administration, but this quickly resulted in concerning rates of Gram-negative resistance [1,2,3]. From 1999 to 2010, *Escherichia coli*’s resistance to ciprofloxacin increased from 3% to >25% in British Columbia [3]. Pathogens causing healthcare-associated and catheter-associated urinary tract infections saw the greatest increase in resistance to fluoroquinolones throughout this time period [2]. In 2016, Talan et al. identified fluoroquinolone resistance rates in the United States among pyelonephritis cases ranging from 6.3% in uncomplicated case to 19.9% in complicated disease [4].

In addition to growing resistance, fluoroquinolone exposure is also associated with a higher risk of infection with *C. difficile* due to alterations in gut microbial flora [5]. Broad-spectrum antibiotics, such as fluoroquinolones, third- and fourth-generation cephalosporins and lincosamides, inhibit growth of intestinal flora leaving patients vulnerable for *C. difficile* infection (CDI). Severe CDI places patients at risk for complications such as pseudomembranous colitis, septic shock and death [6]. Specifically, ribotype 027 is considered to cause serious disease and its spread can be attributed to overuse of fluoroquinolones [7]. The concerning growth in CDI incidence led the Centers for Disease Control and Prevention to classify CDI as an urgent threat in 2019 [8]. Recommendations to reduce CDI incidence include strategies to improve infection control measures and antibiotic use.

The United States Food and Drug Administration (FDA) began adding boxed warnings to fluoroquinolones in 2008, including an increased risk of tendon rupture, peripheral neuropathy and other potentially permanent muscle and central nervous system effects. In 2016, the FDA advised healthcare providers to reserve fluoroquinolones for patients who do not have alternative options for specific indications [9,10]. Based on these disabling and potentially irreversible adverse drug effects, the European Commission issued binding restrictions on fluoroquinolone use in March 2019 [11].

Due to increasing resistance, CDI concerns, and toxicity risks, our health system began focusing on initiatives to reduce unnecessary fluoroquinolone use in 2018 and encouraging utilization of alternative antibiotics where appropriate. The cephalosporins have a similar broad-spectrum of coverage, are available as intravenous and oral formulations, and are utilized in multiple infectious diseases, including many in which fluoroquinolones are commonly used. Relative to fluoroquinolones, cephalosporins are generally more well-tolerated, with oral cephalosporins having fewer adverse events than those administered intravenously [12]. Our health system’s stewardship initiatives involved multiple antibiotic classes, however, we chose to compare fluoroquinolone use versus broad-spectrum cephalosporins based on the similar spectrum of activity and indications for use.

This study aimed to determine if the decrease in fluoroquinolone usage in our healthcare system was associated with an increase in third- and fourth-generation cephalosporin antibiotic use. Secondary outcomes included trends in CDI, changes in rates of resistance to common Gram-negative pathogens, and analyses stratified by antibiotic indications.

## 2. Results

A total of 159 affiliated institutions were included, yielding 2.6 million patients who received either a fluoroquinolone or third- and fourth-generation cephalosporin between June 2018 and July 2021. Of those patients, 34% were female and 29% male, with the remainder unknown. Forty percent of patients were white, non-Hispanic, 11% Hispanic and 9% Black, non-Hispanic. The majority of patients were age 55 and over. Forty-seven percent of patients had a length of stay (LOS) of less than 3 days, 25% had a LOS of 3 to 5 days and 28% had a LOS of more than 5 days. Of the patients who received cephalosporins, 67% received less than 3 days of therapy, 22% received 3 to 5 days of therapy and 11% received greater than 5 days of therapy. In the fluoroquinolone group, 93% of patients received less than 3 days of therapy, 5% received 3 to 5 days of therapy and 2% received greater than 5 days of therapy.

**Figure 1:**
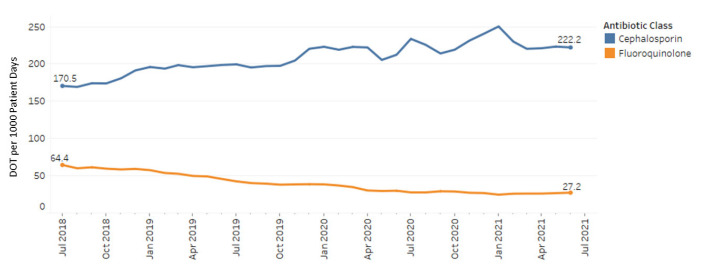
Cephalosporin and fluoroquinolone use July 2018–July 2021.

Throughout the study period, there was a statistically significant decreasing trend in fluoroquinolone use at a rate of 1.18 DOT/1000 PD per month (*p* < 0.001) (Figure 1). This trend was primarily driven by decreases in levofloxacin (0.84 DOT/1000 PD) and ciprofloxacin (0.34 DOT/1000 PD) use. There was no significant change in usage of delafloxacin or moxifloxacin over the study period. Additionally, there was a statistically significant increasing trend of cephalosporin use over the study period of 1.70 DOT/1000 PD per month (*p* < 0.001). Primary drivers of this trend included ceftriaxone (1.20 DOT/1000 PD), cefepime (0.39 DOT/1000 PD) and cefdinir (0.11 DOT/1000 PD). No significant differences were observed in cefixime, cefpodoxime, or ceftazidime.

**Figure 2:**
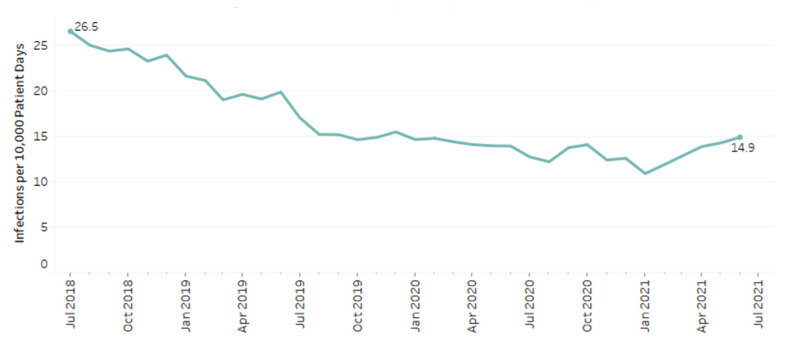
*C. difficile* rates among patients with cephalosporin and fluoroquinolone use.

A total of 24,214 patients had a positive result for *C. difficile,* which amounted to 1% of included patients in this study. At the beginning of the study period, rates of CDI were 26.5 infections per 10,000 patient days compared to 14.9 at the end of the study period (Figure 2). Overall rates of infection decreased throughout the study period by 0.37 infections/10,000 patient days per month (*p* < 0.001).

**Figure 3:**
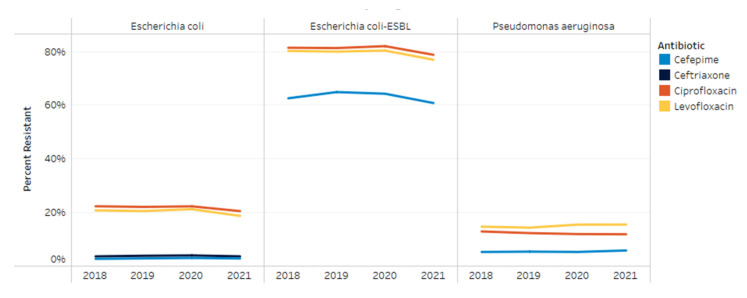
Resistance trends by organism from 2018–2021.

Resistance to ceftriaxone, cefepime, ciprofloxacin and levofloxacin in *E. coli,* extended spectrum beta-lactamase (ESBL)- producing *E. coli* and *P. aeruginosa* are shown in Figure 3. From 2018 to 2021, *E. coli*’s resistance to cefepime and ceftriaxone remained consistent at 3% and 4%, respectively. Resistance to ciprofloxacin and levofloxacin decreased from 22% and 21% to 21% and 19%, respectively, by 2021 (*p* < 0.001; *p* < 0.001). For ESBL *E. coli* isolates, resistance to ciprofloxacin and levofloxacin remained stable from 2018 to 2020 at 82% and 80%, respectively. From 2020 to 2021, ESBL *E. coli*’s resistance to fluoroquinolones decreased to 79% and 77% (*p* < 0.001; *p* < 0.001). Resistance patterns for *P. aeruginosa* did not show any significant changes during the study period for any of the medications included. Resistance among *P. aeruginosa* isolates from 2018 to 2021 to cefepime, ciprofloxacin and levofloxacin was 5–6%, 12–13% and 14–15%, respectively.

**Figure 4:**
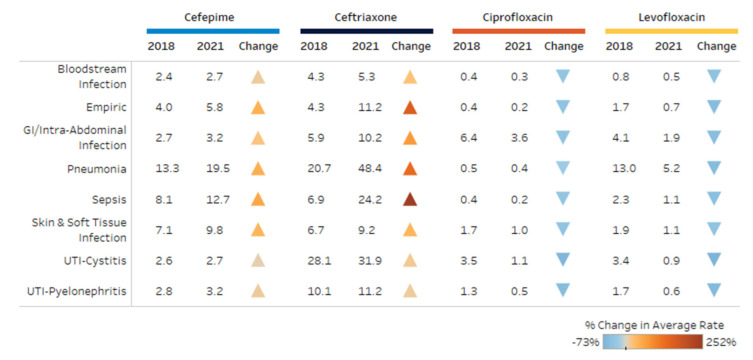
Change in use (DOT per 1000 patient days) for top indications.

The most common indications for ceftriaxone included pneumonia, UTI-cystitis and sepsis. For cefepime, pneumonia, sepsis and skin and soft tissue infections were the most common. Levofloxacin was utilized most often for pneumonia, gastrointestinal (GI)/intra-abdominal infections and UTI-cystitis, while ciprofloxacin was predominantly used for GI/intra-abdominal infections, UTI-cystitis and skin and soft tissue infections. Figure 4 shows the percent change in DOT/1000 PD by indication for each antibiotic during the study period. There was an increase in broad-spectrum cephalosporin use for all indications, whereas fluoroquinolone use decreased for all indications. The largest increases were observed with ceftriaxone for sepsis or empiric use with a greater than 200% increase. Ceftriaxone use for pneumonia increased 2-fold throughout the study period. The highest decrease in ciprofloxacin and levofloxacin were observed with UTI-cystitis and UTI-pyelonephritis.

## 3. Discussion

This study illustrated that a reduction in the use of fluoroquinolones correlated with an increase in use of third- and fourth-generation cephalosporins. This was primarily driven by ceftriaxone and levofloxacin, which was true regardless of indication. Importantly, this change in antibiotic use was associated with a decrease in CDI and improvements in susceptibilities among some Gram-negative pathogens, supporting the notion that antimicrobial stewardship efforts can positively influence susceptibility changes and prevent hospital-acquired infections. The decrease in CDI and improvements in susceptibilities were observed despite a higher percentage of patients in the fluoroquinolone group receiving shorter durations of therapy compared to those in the cephalosporin group. This suggests that even a limited duration of 1 to 2 days of fluoroquinolones can have detrimental effects on our patients.

Our study’s findings are consistent with what has been reported previously. A 2019 study by Redmond et al. found that decreased prescribing of fluoroquinolones along with implementation of an environmental cleaning initiative over a 10-year period was associated with decreased healthcare-associated CDI rates in a hospital and long-term care facility [13]. Although cephalosporin antibiotics are also associated with a higher risk of CDI, their study observed a non-statistically significant increase in cephalosporin use while CDI rates declined [13]. Shea et al. identified decreased rates of CDI in a small health-system following restriction of fluoroquinolone use to approved indications [14]. Public health interventions in the United Kingdom have shown similar associations with restricted fluoroquinolone use and decreased CDI rates [15].

This study has several limitations. The effects of unmeasured confounders such as changes in antibiotic stewardship initiatives, advances in infection prevention strategies or the challenges posed by the COVID-19 pandemic may all play a role in the outcomes of this study. In particular, implementation of new antibiotic stewardship initiatives and improvements in infection prevention strategies would also reduce CDI and antibiotic resistance. The ongoing COVID-19 pandemic led to fewer individuals seeking healthcare, increased patient isolation in the acute care setting and heightened infection control measures. These collective behaviors from the pandemic may have resulted in improvements in CDI rates in our healthcare system. The COVID-19 pandemic also influenced antimicrobial use when increased numbers of patients presented with suspected cobacterial pneumonia. As shown in Figure 1, there was increased utilization of antibiotics during waves of the pandemic, which largely impacted the use of cephalosporins. Several published studies described a decrease in CDI rates during the pandemic which was attributed to infection prevention measures and isolation as well as antibiotic choice for presumed cobacterial pneumonia [16,17]. Another limitation was that data were matched with a third-party clinical surveillance software, and all demographic data were not available. This led to some baseline demographic information missing, which limits generalizability of our findings, since the full spectrum of our patient population was unknown. In evaluating CDI rates, only patients who received fluoroquinolones or broad-spectrum cephalosporins were included, limiting the extrapolation of data to other antibiotic trends or stewardship interventions.

Other classes of antibiotics such as carbapenems, lincosamides and penicillins are also associated with a risk of CDI, but they were not measured in this study [18,19]. Fluoroquinolones and broad-spectrum cephalosporins are some of the most highly implicated antibiotics contributing to CDI, and they are some of the most commonly used antibiotics in our healthcare system. As a result, we believe that patterns in use of other antibiotics would likely have less of an impact on outcomes evaluated in this study. However, it has been shown that antibiotic use in patients can have an indirect effect on CDI by augmenting the risk of transmission and infection to unexposed patients in a similar ward or unit, which we did not measure [20,21]. Additionally, we were not able to account for patients who may have been readmitted with *C. difficile* after exposure to a fluoroquinolone or cephalosporin during a prior admission. By only evaluating CDI among patients who received fluoroquinolones or third- and fourth-generation cephalosporins during that same admission, we may have underestimated the true impact of decreased fluoroquinolone use on *C. difficile* rates. Prior studies have also focused on specific strains of CDI such as the epidemic ribotype 027, which is notable for fluoroquinolone-resistance [7,13,22]. This study did not include reporting of specific strains of CDI, limiting our ability to comment on trends in ribotype 027. Another limitation of our study is that we did not evaluate duration of therapy, utilization of oral versus intravenous formulations or other stewardship initiatives specific to reducing fluoroquinolone use that may impact CDI rates or antibiotic resistance. Lastly, it is important to note that our data were unable to determine appropriateness of antibiotic use. While reducing unnecessary fluoroquinolone use was a targeted stewardship strategy in our healthcare system, there are some patients in which fluoroquinolones may be an optimal choice based on the indication and infecting pathogen. Despite these limitations, strengths of our study include the large sample size and the ability to evaluate a diverse patient population across the United States.

## 4. Materials and Methods

This retrospective descriptive analysis was approved by the University of Tennessee Health Science Center Investigational Review Board. Patient data were collected through clinical surveillance software and electronic medical records for all adult patients discharged from an inpatient facility within the HCA Healthcare enterprise utilizing computerized provider order entry between July 2018 and June 2021. HCA Healthcare consists of over 180 hospitals in 20 states and the United Kingdom. Patients included were of racially diverse backgrounds in a variety of settings throughout the health-system.

Antibiotic use data were collected using days of therapy per 1000 patient-days. Patients were included if they received a fluoroquinolone or a third- and fourth-generation cephalosporin. Fluoroquinolones were defined as ciprofloxacin, levofloxacin, moxifloxacin or delafloxacin, and broad-spectrum cephalosporins (third- and fourth-generation) were defined as cefdinir, cefixime, cefotaxime, cefpodoxime, ceftriaxone, ceftazidime or cefepime. Positive results for CDI and fluoroquinolone- and cephalosporin-susceptibility data for *E. coli* and *Pseudomonas aeruginosa* isolates were collected, when available, from the electronic health record in included patients during the same admission. *C. difficile* rates were expressed as infections per 10,000 patient-days among patients included in the study. Trends over time for antibiotic use and *C. difficile* rates were examined using linear regression analyses, with beta coefficients representing the change in rate per month. Differences in antibiotic resistance between the years were analyzed with Chi-squared tests. All statistical analyses and visualizations were done using R (R Foundation; Vienna, Austria) and Tableau (Tableau Software; Seattle, WA, USA).

## 5. Conclusions

In conclusion, targeting unnecessary fluoroquinolone use across a large healthcare system led to improvements in susceptibilities and a reduction in CDI despite increases in broad-spectrum cephalosporins. Although positive outcomes were observed, the findings from this study also suggest there may be opportunities for increased prescribing of narrower-spectrum antibiotics, such as aminopenicillins or first- and second-generation cephalosporins. Additionally, there may be opportunities to reduce fluoroquinolone orders, since a limited exposure of just 1–2 days may still have negative impacts.

Facilities that implement strategies to decrease fluoroquinolone use should monitor fluoroquinolone orders (versus days of therapy), third- and fourth-generation cephalosporin use, and maximize the use of narrower-spectrum antibiotics as appropriate. Future research is warranted to determine the implications of increased broad-spectrum cephalosporin use on antimicrobial resistance and CDI rates as cephalosporins have also been associated with an increased risk for CDI [14]. Further study may also be needed to increase generalizability to other health systems and incorporate other recommended antimicrobial stewardship outcomes associated with changes in prescribing practices [23].

## Data Availability

The data presented in this study are available on request from the corresponding author. The data are not publicly available due to privacy.
